# Suppurating Myoma of the Uterine Wall Followed by Twin Pregnancy

**Published:** 1894-04

**Authors:** James F. W. Ross

**Affiliations:** Lecturer in Gynecology in the Woman’s Medical College; Gynecologist to St. John’s Hospital, Toronto General Hospital, and St. Michael’s Hospital


### Selections.

### SUPPURATING MYOMA OF THE UTERINE WALL FOLLOWED BY TWIN PREGNANCY.

By JAMES F. W. ROSS, M. D.
Lecturer in Gynecology in the Woman's Medical College ; Gynecologist to St. John's Hospital, Toronto General Hospital, and St. Michael's Hospital.

THE following notes are taken from my case book : Mrs. J., æt. 28, kindly referred to me by Dr. Stevenson, of Trenton, admitted July 22, 1891. Nothing unusual about menstrual history ; was married two years ago ; had a child born in May, 1891, good delivery. Four years ago she had a sickness that was attended by much vomiting; had a good deal of pain in the region of the bladder and pain in passing water—only a few drops of water would pass at a time, and there was a frequent desire to micturate. On the 27th of May her child was born ; four days after she noticed a pain in the right iliac region, that shot across the abdomen and across the back ; the pain was severe. At first it was constant ; it gradually became diminished ; and now, July 22d, only occurs for a few hours every day or night. Since her confinement she has noticed an enlargement on the right side, low down in the abdomen. After the confinement the lochial discharge lasted for six weeks.

Two weeks ago—that is, during the first week in July, and about five weeks after her confinement—a greenish-yellow, thick foul-smelling discharge commenced to flow freely from the vagina. At present the discharge is thinner, not so offensive, and has every appearance of laudable pus. The discharge has gradually been increasing in quantity. Patient looks very ill ; she lies on her left side, as it hurts her to turn on her back. There is pain on pressure over the abdomen, chiefly in the left iliac region ; tympanites present ; she has an aching pain in the right iliac region. Has been troubled with painful micturition for the last week or two. The urine is found normal.

On inspecting the genitals, I found a large abscess in the left labia majora, and a very offensive discharge of pus from the vagina ; the parts were all reddened and inflamed as a consequence of the irritation of the discharge of this pus. The os uteri was enlarged, and the cervical canal patulous. One finger was passed in, and a large fibroid, about the size of a child's head, was discovered pressing into the cavity of the uterus. The patient was placed under chloroform, and on exploring the interior of the uterus the fingers burst into a horrible sloughing mass, evidently a sloughing intramural fibroid. The cervix was still further dilated, and by means of gallstone forceps and fingers the tumor was scooped out until the surface towards the peritoneal cavity was as much diminished in thickness as was consistent with safety. The hemorrhage was free, but the uterine cavity, and the cavity of the tumor, were tamponed tightly with iodoform gauze tied into knots, together with vaginal packing, and a pad and bandage to compress the genitals.

The abscess in the left labia majora was freely incised, and a large quantity of pus evacuated. The uterine cavity was douched out twice daily for a few days, and the uterine cavity repacked with iodoform gauze. The patient made an uninterrupted recovery. One would have thought that the uterus of such a case would be so much weakened by such a large growth in its wall that pregnancy would scarcely be carried through at any subsequent period with safety. The tumor had a very broad attachment, and was not one of those intra-uterine fibroids with a pedicle, but bulged out into the abdominal cavity, so that the junction of the uterine muscular tissue could be distinctly felt above and below it by a depression. The uterine wall was implicated from the fundus to the internal os.

The reason I relate the history of the case at this late date after her recovery is owing to the fact that I have just received a letter from her medical attendant, who states: "Mrs. J., the patient of mine on whom you operated, has since been safely delivered of twins. She made a good recovery, and is in good health."—*Canadian Practitioner*, February, 1894.

